# Age-based factors modulating the required thyroxine dose to achieve thyrotropin suppression in intermediate-and high-risk papillary thyroid cancer

**DOI:** 10.3389/fendo.2023.1126592

**Published:** 2023-06-14

**Authors:** Ruo-Yun Zhou, Ning Li, Hai-Long Tan, Neng Tang, Pei Chen, Mian Liu, Deng-Jie Ou-Yang, Zi-En Qin, Lei Ai, Bo Wei, Ya-Xin Zhao, Shi Chang, Peng Huang

**Affiliations:** ^1^ Department of General Surgery, Xiangya Hospital Central South University, Changsha, Hunan, China; ^2^ Clinical Research Center for Thyroid Disease in Hunan Province, Xiangya Hospital Central South University, Changsha, Hunan, China; ^3^ Hunan Provincial Engineering Research Center for Thyroid and Related Diseases Treatment Technology, Xiangya Hospital Central South University, Changsha, Hunan, China; ^4^ National Clinical Research Center for Geriatric Disorders, Xiangya Hospital, Changsha, Hunan, China

**Keywords:** thyrotropin suppression, intermediate-and high-risk differentiated thyroid cancer, individualized medication regimens, age stratification, thyroid cancer

## Abstract

**Background:**

Guidelines widely recommend thyrotropin suppression to reduce the risk of recurrence in intermediate- and high-risk papillary thyroid cancer (PTC) after total thyroidectomy. However, an insufficient or excessive dosage may result in a number of symptoms/complications especially in older patients.

**Patients and methods:**

We constructed a retrospective cohort including 551 PTC patient encounters. Using propensity score matching and logistic regression models, we determined the independent risk factors affecting levothyroxine therapy at different ages. Our outcomes included: expected TSH level and an unexpected TSH level, which was based on the initial thyroid-stimulating hormone (TSH) goal< 0.1 mIU/L with usual dosage of L-T4 (1.6 μg/kg/day).

**Results:**

From our analysis, more than 70% of patients undergoing total thyroidectomy did not achieve the expected TSH level using an empirical medication regimen, and the effect of the drug was affected by age (odds ratio [OR], 1.063; 95% CI, 1.032-1.094), preoperative TSH level (OR, 0.554; 95% CI, 0.436-0.704) and preoperative fT3 level (OR, 0.820; 95% CI, 0.727-0.925). In patients with age < 55 years old, preoperative TSH level (OR, 0.588; 95% CI, 0.459-0.753), and preoperative fT3 level (OR, 0.859; 95% CI, 0.746-0.990) were two independent protective factors, while, in patients with age ≥ 55 years old, only preoperative TSH level (OR, 0.490; 95% CI, 0.278-0.861) was the independent protective factors to achieve expected TSH level.

**Conclusion:**

Our retrospective analysis suggested the following significant risk factors of getting TSH suppression in PTC patients: age (≥55 years), lower preoperative TSH and fT3 levels.

## 1 Introduction

Over the past several decades, there has been an increase in the incidence of thyroid cancer, with the vast majority being papillary thyroid cancer (PTC) (1). Although the 5-year survival rate of PTC is 90% or more, the risk of cancer recurrence is 5-20% or even higher in patients judged to be at intermediate to high risk. Total thyroidectomy is recommended by the American Thyroid Association (ATA, 2015 edition) to reduce the risk of recurrence (2). After total thyroidectomy, levothyroxine (LT4) is prescribed to replace the function of the removed thyroid gland. Meanwhile, a supraphysiological dose of LT4 is applied to suppress the secretion of thyroid-stimulating hormone (TSH), which further reduces the risk of recurrence (3, 4).

For patients with different levels of predicted risks, different postoperative TSH targets are recommended. In addition, the benefits of TSH suppression treatment could be outweighed by the side effects as age advances. It is widely acknowledged that iatrogenic thyrotoxicosis resulting from aggressive suppression therapy is associated with an increased risk of fracture and cardiovascular events (5, 6), 2- to 4-fold higher in elderly patients (7, 8). Organ declines and adaptive changes in the hypothalamic-pituitary-thyroid axis of aged individuals, who are at high risk of thyroid cancer (2, 9) and consequently the main group receiving thyroidectomy (1), complicate the selection of the individual dosage of LT4. Even though optimal TSH goals were set to balance the adverse effects of TSH suppression and recurrence risk, individual variations in responses to LT4 remain unresolved, and there is no appropriate LT4 dosage prediction strategy for patients, especially for the aged.

In clinical routine, patients are usually prescribed an initial dosage LT4 of 1.6 μg/kg/day at the beginning of suppression therapy (10). However, the required LT4 dosage is affected by more than just body weight (BW). It has been suggested that body mass index (BMI) and body surface area (BSA) may predict the initial dosage more effectively (11, 12). Sex, age, and autoimmune diseases have also been reported to affect the LT4 requirement (13–16). Drugs and comorbidities can interfere with the treatment effect by disturbing the absorption or metabolism of LT4 (17–19). In addition, LT4 requirements are apparently affected by environmental temperature since thyroid hormones play an important role in regulating metabolism. The metabolism rate and function of the endocrine system vary with advancing age (20, 21), which accounts for different responses to LT4 in people of different ages. It is necessary to develop a specific strategy for LT4 dosage selection for elderly patients.

In this study, we sought to identify the effect that age has on the treatment efficiency of LT4. Patients undergoing initial total thyroidectomy with intermediate to high risk were recruited and treated with LT4 following the guidance of the American Thyroid Association (ATA, 2015 edition) (2). We conducted propensity score matching (PSM) to diminish the effect of BW and ambient temperature. Other characteristics of the LT4-sensitive groups were explored, including age, sex, BMI, BSA, thyroid thickness, preoperative thyroid function, Hashimoto’s thyroiditis (HT), hypertension and diabetes mellitus to offer a theoretical basis for individual medicine care during suppression therapy.

## 2 Materials and methods

### 2.1 Patients and procedures

This study is a single-center retrospective analysis that received approval from the medical ethics committee at Xiangya Hospital (20211245). Patients who met the following requirements were included in the study: (1) initially undergoing total thyroidectomy for thyroid cancer with an intermediate or high risk for recurrence (ATA, 2015 edition) (2); (2) taking LT4 per day based on the 1.6 µg/kg dosing regimen; (3) clinicopathologic data complete; and (4) no thyroid hormones or iodine given before surgery. The cohort was followed from January 2016 to October 2019 at the Department of General Surgery, Xiangya Hospital of Central South University.

A total of 551 patients met the criteria and were enrolled in this study. All the patients completed a physical examination, thyroid function test, ultrasonography of the thyroid, and measurements of serum thyroid peroxidase antibodies (TPOAb) and anti-thyroglobulin antibodies (TgAb) before surgery and were followed up at 1 month for postoperative TSH measurement. All tests used immunochemiluminometric assays conducted at the same laboratory. The reference ranges are 2.8-7.1pmol/l for serum free T3 (fT3), 12~22pmol/l for serum free T4 (fT4), and 0.27-4.2mIU/L for TSH.

According to the American Thyroid Association, initial TSH suppression to below 0.1 mIU/L is recommended among thyroid cancer patients at high risk of recurrence (ATA, 2015 edition) (2). At the first follow-up after surgery, the TSH suppression treatment effect was classified into the expected level or an unexpected level, corresponding to a serum TSH level <0.1 mIU/L and ≥0.1 mIU/L. We further divided the unexpected level into two groups, the replacement group and the hypothyroidism group, and the corresponding TSH levels were 0.1~4.2 mIU/L and ≥4.2 mIU/L, respectively.

### 2.2 Clinicopathological characteristic

To investigate the clinicopathological characteristics of patients who obtained different treatment effects, the variables collected included age, sex, BMI, BSA, average temperature during operation month, preoperative serum TSH, preoperative serum fT3, preoperative serum fT4, thyroid thickness, HT, and comorbidities including hypertension and diabetes mellitus. The mean temperature during the operative month is available on the website of the China Meteorological Administration. The thyroid thickness was calculated by the average of the left and right glands obtained from the preoperative ultrasound report.

### 2.3 Statistical analysis

SPSS 26.0 software was used to conduct the statistical analyses. Continuous data are presented as the mean and standard deviation (SD), and the Mann−Whitney U test was used for comparisons between groups. The binary data are expressed as the patient number with their percentages, and the chi square test was used for comparisons between two groups. PSM was performed by using the nearest-neighbor matching method with a caliper distance of 0.02 without replacement between chosen groups. Weight and ambient temperature were used to calculate the propensity score to perform the matching. Significant variables with P <0.05 after PSM were entered into the multivariate binary logistic regression analysis to identify the factors affecting the treatment outcome. The effect of the factors was presented with the OR and 95% confidence interval (CI). A level of P<0.05 was used to indicate significance, and all statistical tests were two-tailed. A receiver operating characteristic curve (ROC) analysis was carried out to quantify the prediction performance of the identified factors, and the value of the area under the ROC curve (AUC) was calculated.

## 3 Results

### 3.1 More than 70% of patients did not achieve the expected TSH level using an empirical medication regimen

We selected 551 patients from the database who took LT4 (Euthyrox, Merck KGaA) at 1.6 μg/kg per day after total thyroidectomy, including 425 women (76.2%) and 126 men (23.8%) at a ratio of 3.4:1. Their mean age was 43.93 ± 11.18 years, ranging from 18 to 73 years. The average weight, height, BMI, and BSA of the 551 patients were 61.61 ± 10.48 kg, 161.05 ± 6.75 m, 23.70 ± 3.26 kg/m^2^, and 1.66 ± 0.16 m2, respectively. Preoperative thyroid function included TSH (3.01 ± 2.57 mIU/L), fT3 (7.12 ± 5.02 pmol/L), and fT4 (13.67 ± 6.44 pmol/L). The average thyroid thickness was 16.38 ± 4.04 mm before surgery. Hashimoto’s thyroiditis, hypertension and diabetes mellitus were found in 127 patients (23%), 75 patients (13.6%), and 21 patients (3.8%), respectively (**Table 1**).

**Table 1:** Demographics and clinical characteristics of 551 PTC patients who underwent total thyroidectomy.

Variables	Total	Expected level	Unexpected level			*p*
			Total	Replacement	Hypothyroidism	
	n (%)/mean ± SD	n (%)/mean ± SD	n (%)/mean ± SD	n (%)/mean ± SD	n (%)/mean ± SD	
Number	551 (100)	130 (23.6)	421 (76.4)	258 (46.8)	163 (29.8)	
Sex, Female	425 (76.2)	123 (94.4)	302 (71.7)	201 (77.9)	101 (61.8)	**<0.001^a^**
Age, year	43.93 ± 11.18	47.80 ± 11.06	42.73 ± 10.96	42.78 ± 10.71	42.67 ± 11.18	**<0.001^b^**
Height, m	161.05 ± 6.75	158.12 ± 5.53	161.95 ± 6.84	161.25 ± 6.67	163.06 ± 6.97	**<0.001^b^**
Weight, kg	61.61 ± 10.48	56.33 ± 6.99	63.24 ± 10.84	62.36 ± 10.18	64.62 ± 11.71	**<0.001^b^**
BSA, m^2^	1.66 ± 0.16	1.57 ± 0.11	1.68 ± 0.17	1.67 ± 0.16	1.71 ± 0.18	**<0.001^b^**
BMI	23.70 ± 3.26	22.53 ± 2.60	24.06 ± 3.36	23.96 ± 3.33	24.20 ± 3.42	**<0.001^b^**
Thyroid thickness, mm	16.38 ± 4.04	16.22 ± 4.37	16.43 ± 3.94	16.27 ± 3.80	16.68 ± 4.14	0.202^b^
Ambient temperature, °C	17.33 ± 8.93	20.38 ± 7.65	16.39 ± 9.10	19.10 ± 8.38	12.11 ± 8.55	**<0.001^b^**
Preoperative TSH, mIU/L	3.01 ± 2.57	1.84 ± 1.31	3.37 ± 2.76	2.78 ± 1.92	4.30 ± 3.52	**<0.001^b^**
Preoperative fT3, pmol/L	7.12 ± 5.02	4.92 ± 1.73	7.82 ± 5.48	5.46 ± 2.97	11.56 ± 6.40	**<0.001^b^**
Preoperative fT4, pmol/L	13.67 ± 6.44	16.12 ± 4.02	12.91 ± 6.85	15.58 ± 4.67	8.69 ± 7.62	**0.013^b^**
Post-operative fT3, pmol/L	8.44 ± 7.60	5.76 ± 1.34	9.27 ± 8.49	5.66 ± 3.84	14.99 ± 10.48	**<0.001^b^**
Post-operative fT4, pmol/L	17.48 ± 9.12	24.94 ± 4.56	15.18 ± 8.95	20.08 ± 5.11	7.42 ± 8.21	**<0.001^b^**
Type II diabetes						
With	21 (3.8)	3 (2.4)	18	11 (4.2)	7 (3.3)	<0.306^a^
Without	530 (96.2)	127 (97.6)	247	247 (95.8)	156 (96.7)	
Hypertension						
With	75 (13.6)	15 (12.1)	60	42 (15.8)	18 (11.2)	<0.430^a^
Without	476 (86.4)	115 (87.9)	361	216 (84.2)	145 (88.8)	
Hashimoto’s thyroiditis						
With	127 (23.0)	24 (17.7)	103	63 (23.3)	40 (25.0)	<0.155^a^
Without	424 (77.0)	106 (82.3)	318	195 (76.7)	123 (75.0)	

Participants were grouped according to the TSH levels at 1 month on suppression therapy with a daily L-T4 dose of at least 1.6 μg/kg after total thyroidectomy. The expected level was defined as TSH levels below physiological levels (<0.1mIU/L), the replacement group was TSH levels equal to physiological levels (0.1~4.2 mIU/L), and the hypothyroidism group was defined as TSH levels above physiological levels (≥4.2 mIU/L). Variables with statistical significance are shown in bold, ^a^ chi-square test, ^b^ Mann−Whitney test.

The detailed characteristics of the different treatment effects are summarized in **Table 1**. A total of 23.6% of patients had their TSH suppressed under 0.1 mIU/L, 46.8% of the patients were euthyroid, and 29.8% were hypothyroid at the first follow-up. There were significant differences between the two groups with different TSH suppression levels, including age, sex, height, weight, BMI, BSA, ambient temperature, preoperative serum fT3 and preoperative serum TSH (all p<0.001), and preoperative serum fT4(p=0.013). Among patients who obtained the expected level, women distinctly made up the majority compared with patients who had an unexpected level (expected level 94.4% women vs. unexpected level 71.7% women). Patients in the unexpected level group had higher fT3 (expected level 4.92 ± 1.73 pmol vs. unexpected level 7.82 ± 5.48 pmol) and higher TSH (expected level 1.84 ± 1.31 mIU/L vs. unexpected level 3.37 ± 2.76 mIU/L) and lower fT4 (expected level 16.12± 4.02 pmol/l vs. unexpected level 12.91 ± 6.85 pmol/l). Their weight showed an increasing trend, while ambient temperature showed a decreasing trend as the treatment effect weakened. There were no significant differences in the categorical data except for sex between the two levels (diabetes mellitus, p = 0.306; hypertension, p = 0.430; Hashimoto’s thyroiditis, p = 0.155) (**Table 1**).

### 3.2 Age, preoperative TSH level and fT3 level were independent risk factors after PSM

Ambient temperature (p <0.001) and weight (p <0.001) had significant differences between the expected level and unexpected level before PSM. All the other variables also showed significant differences between the two levels. After 1:1 PSM, weight and mean temperature reached an equilibrium (weight, p =0.338; ambient temperature, p = 0.236). Sex and BSA were not significantly different between the matched groups, while age, height, BMI and preoperative fT3, fT4, and TSH remained significantly different between the two groups. Age seemed to have a higher possibility of being different between the two groups (p<0.001).

**Figure 1:**
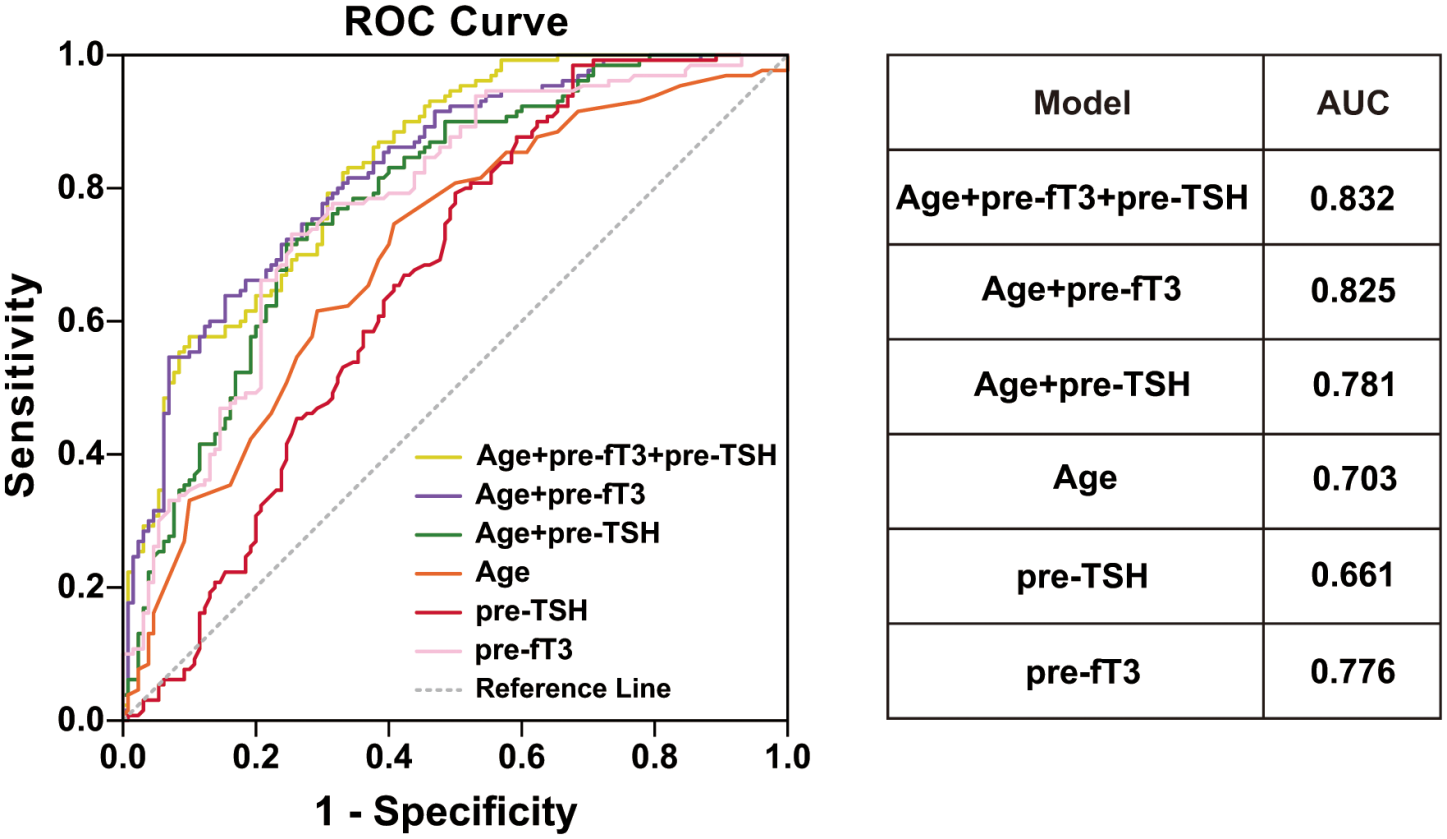
ROC curve and AUC for different model. age (orange line), preoperative TSH (red line), preoperative fT3 (pink line), age+preoperative fT3 (purple line), age+preoperative TSH (green line), age+preoperative TSH +preoperative fT3 (yellow line).

We further explored the potential prognostic factors for obtaining the expected level using univariate binary logistic regression analysis. Age (p<0.001, OR=1.063, 95% CI, 1.032-1.094), preoperative TSH (p<0.001, OR=0.554, 95% CI, 0.436-0.704) and preoperative fT3 (p=0.001, OR=0.820, 95% CI, 0.727-0.925) were all significantly associated with the treatment effect (**Table 2**). ROC analyses were performed to quantify the prediction performance of the three identified factors. Preoperative fT3 was identified as the most effective prediction factor, with an AUC of 0.776, followed by age, with an AUC of 0.703. Using age and preoperative fT3 as co-prediction factors had the highest AUC of 0.825 (**Figure 1**).

**Table 2:** Variables before and after PSM between the expected and unexpected level.

Variables	Before PSM			After PSM			p*	OR (95%CI)
	Unexpected level (n=421)	Expected level (n=130)	*p*	Unexpected level (n=130)	Expected level (n=130)	*p*		
Weight, kg	63.24 ± 10.84	56.33 ± 6.99	<**0.001** ^b^	55.32 ± 6.07	56.33 ± 6.99	0.338^b^		
Ambient temperature, °C	16.39 ± 9.10	20.38 ± 7.65	<**0.001** ^b^	18.52 ± 9.24	20.38 ± 7.65	0.236^b^		
Age	42.73 ± 10.96	47.80 ± 11.06	<**0.001** ^b^	39.74 ± 11.19	47.80 ± 11.06	<**0.001** ^b^	<**0.001**	1.063 (1.032,1.094)
Sex								
Male	119 (28.3%)	7 (5.4%)	<**0.001** ^a^	14 (10.8%)	7 (5.4%)	0.111^a^		
Female	302 (71.7%)	123 (94.6%)		116 (88.2%)	123 (94.6%)			
Height, m	161.95 ± 6.84	158.12 ± 5.53	<**0.001** ^b^	159.65 ± 5.03	158.12 ± 5.53	**0.020** ^b^	0.137	
BMI	24.06 ± 3.36	22.53 ± 2.60	<**0.001** ^b^	21.71 ± 2.19	22.53 ± 2.60	**0.010^b^**	0.372	
BSA, m^2^	1.68 ± 0.17	1.57 ± 0.11	<**0.001** ^b^	1.56 ± 0.10	1.57 ± 0.11	0.791^b^		
Preoperative TSH, mIU/L	3.37 ± 2.76	1.84 ± 1.31	<**0.001** ^b^	3.93 ± 3.95	1.84 ± 1.31	<**0.001** ^b^	<**0.001**	0.554 (0.436,0.704)
Preoperative fT3, pmol/L	7.82 ± 5.48	4.92 ± 1.73	<**0.001** ^b^	8.62 ± 6.01	4.92 ± 1.73	<**0.001** ^b^	**0.001**	0.820 (0.727,0.925)
Preoperative fT4, pmol/L	12.91 ± 6.85	16.12 ± 4.02	<**0.001** ^b^	12.09 ± 7.95	16.12 ± 4.02	**0.006^b^**	0.579	

Variables with statistical significance were shown in bold. Weight and mean temperature were matched between groups. ^a^ chi-square test, ^b^ Mann−Whitney test. p* was the result of binary logistic regression analysis.

### 3.3 Male sex and preoperative fT3 levels were independent risk factors leading to inadequate efficacy of LT4 dosage

Between the replacement group and hypothyroid group, ambient temperature showed a significant difference (p<0.001), while weight was already equilibrated (p=0.065). After 1:1 PSM, the ambient temperature reached equilibrium. Other variables, including sex, height, BSA, BMI, preoperative serum TSH, preoperative serum fT3 and preoperative serum fT4 (all p<0.001), were significantly different before PSM. After PSM, only sex, preoperative serum TSH, preoperative serum fT3 and preoperative serum fT4 (all p<0.001) were distributed differently between the replacement and hypothyroidism groups. The multivariate binary logistic regression analysis showed that sex and preoperative fT3 were the two factors that affected the treatment effect. Male sex (p<0.001, OR=3.248, 95% CI, 1.708-6.177) and patients with higher preoperative fT3 (p=0.001, OR=1.248, 95% CI, 1.101-1.158) were more likely to be in above-target TSH state (**Table 3**).

**Table 3:** Variables before and after PSM between replacement and hypothyroidism groups.

Variables	Before PSM			After PSM			p*	OR (95%CI)
	Replacement (n=258)	Hypothyroidism (n=163)	*p*	Replacement (n=116)	Hypothyroidism (n=116)	*p*		
Weight, kg	62.36 ± 10.18	64.62 ± 11.71	0.065^b^	62.54 ± 10.18	63.99 ± 12.25	0.356^b^		
Ambient temperature, °C	19.10 ± 8.38	12.11 ± 8.55	**<0.001^b^**	14.10 ± 7.75	14.46 ± 8.93	0.864^b^		
Age	42.78 ± 10.71	42.67 ± 11.18	0.866^b^	42.45 ± 10.76	41.53 ± 11.62	0.423^b^		
Sex								
Male	57 (22.1%)	62 (38.0%)	<**0.001** ^a^	25 (21.6%)	42 (36.2%)	**0.014^a^**	**<0.001**	3.248 (1.708,6.177)
Female	201 (77.9%)	101 (62.0%)		91 (78.4%)	74 (63.8%)			
Height, m	161.25 ± 6.67	163.06 ± 6.97	<**0.001** ^b^	161.31 ± 6.08	163.15 ± 7.00	0.059^b^		
BMI	23.96 ± 3.33	24.20 ± 3.42	<**0.001** ^b^	24.05 ± 3.22	23.92 ± 3.60	0.710^b^		
BSA, m^2^	1.66 ± 0.16	1.71 ± 0.18	<**0.001** ^b^	1.67 ± 0.15	1.70 ± 0.19	0.276^b^		
Preoperative TSH, mIU/L	2.78 ± 1.92	4.30 ± 3.52	**<0.001^b^**	2.90 ± 2.26	4.14 ± 4.10	<**0.001** ^b^	0.148	
Preoperative fT3, pmol/L	5.46 ± 2.97	11.56 ± 6.40	**<0.001^b^**	5.84 ± 3.60	10.03 ± 6.27	<**0.001** ^b^	**0.001**	1.248 (1.101,1.158)
Preoperative fT4, pmol/L	15.58 ± 4.67	8.69 ± 7.62	**<0.001^b^**	14.80 ± 4.79	10.51 ± 7.58	**0.001** ^b^	0.370	

Variables with statistical significance are shown in bold. Weight and mean temperature were matched between groups. ^a^ chi-square test and ^b^ Mann−Whitney test. p* was the result of binary regression.

### 3.4 Patients with low preoperative TSH and fT3 require lower dosage if aged under 55 years old

It has now been established that age is a factor that influences whether the expected TSH level is met, and we identified additional factors that influence the treatment effect of LT4 in different age groups. We divided the patients into two age groups based on age, using 55 years as the cut-off value according to the 8th American Joint Committee on Cancer (AJCC) staging system (18). We also conducted PSM before multivariate binary logistic regression. In the group under 55 years old, sex, height, BMI, preoperative serum TSH and preoperative fT3 were included in the multivariate regression analysis. Only preoperative serum TSH (p<0.001, OR=0.588, 95% CI, 0.459-0.753) and preoperative fT3 (p=0.035, OR=859, 95% CI, 0.746-0.990) were significantly different (**Table 4**).

**Table 4:** Variables before and after propensity score matching among patients under 55 years old.

Variables	Before PSM			After PSM			p*	OR (95%CI)
	Unexpected level (n=358)	Expected level (n=95)	*p*	Unexpected level (n=95)	Expected level (n=95)	*p*		
Weight, kg	62.74 ± 10.93	56.27 ± 7.40	**<0.001^b^**	55.64 ± 8.18	56.27 ± 7.40	0.322^b^		
Ambient temperature, °C	16.57 ± 9.07	20.24 ± 7.80	**<0.001^b^**	19.13 ± 8.97	20.24 ± 7.80	0.634^b^		
Sex								
Male	97 (27.1%)	4 (4.2%)	<**0.001** ^a^	15 (15.8%)	4 (4.2%)	**0.008^a^**	0.114	
Female	261 (72.9%)	91 (95.8%)		80 (84.2%)	91 (95.8%)			
Height, m	162.06 ± 6.87	158.66 ± 5.46	<**0.001^b^**	160.63 ± 5.82	158.66 ± 5.46	**0.034^b^**	0.253	
BMI	23.82 ± 10.84	22.34 ± 6.63	<**0.001^b^**	21.55 ± 8.12	22.34 ± 6.63	**0.009^b^**	0.136	
BSA, m^2^	1.68 ± 0.17	1.57 ± 0.12	<**0.001^b^**	1.57 ± 0.13	1.57 ± 0.12	0.707^b^		
Preoperative TSH,mIU/L	3.38 ± 2.90	1.87 ± 1.28	**<0.001^b^**	3.69 ± 4.35	1.87 ± 1.28	<**0.001** ^b^	<**0.001**	0.588 (0.459,0.753)
Preoperative fT3, pmol/L	7.72 ± 5.47	4.98 ± 1.89	**<0.001^b^**	7.36 ± 5.26	4.98 ± 1.89	<**0.001** ^b^	**0.035**	0.859 (0.746,0.990)
Preoperative fT4, pmol/L	13.20 ± 6.82	16.14 ± 4.23	**0.092^b^**	14.23 ± 7.30	16.14 ± 4.23	0.960^b^		

Variables with statistical significance are shown in bold. Weight and mean temperature were matched between groups. ^a^ chi-square test, ^b^ Mann−Whitney test. p* was the result of binary regression analysis.

### 3.5 Patients with low preoperative TSH require lower dosage if aged 55 and older

In the group 55 years of age and older, all of the variables were significantly different between the unexpected level and expected level groups before PSM. After PSM, weight (p=0.775) and ambient temperature (p=0.870) were equilibrated. Preoperative TSH was slightly higher after PSM 3.57 ± 2.10 mIU/L vs. before PSM 3.27 ± 1.74 mIU/L in the unexpected level group after PSM and was the only variable that remained significantly different after PSM (p=0.010). In binary logistic analysis, preoperative TSH was an independent protective factor (p=0.013, OR=0.490, 95% CI, 0.278-0.861) (**Table 5**).

**Table 5:** Variables before and after PSM among patients aged 55 and older.

Variables	Before PSM			After PSM			p*	OR (95%CI)
	Unexpected level (n=63)	Expected level (n=35)	*p*	Unexpected level (n=15)	Expected level (n=15)	*p*		
Weight, kg	66.07 ± 9.97	56.47 ± 5.82	**<0.001^b^**	59.17 ± 6.96	58.60 ± 6.60	0.775^b^		
Ambient temperature, °C	15.38 ± 9.27	20.77 ± 7.35	**0.006^b^**	18.77 ± 8.25	19.60 ± 8.28	0.870^b^		
Sex								
Male	22(34.92%)	3(8.6%)	**0.004** ^a^	4(15.8%)	2(4.2%)	0.361^a^		
Female	41(65.08%)	32(91.4%)		11(84.2%)	13(95.8%)			
Height, m	161.32 ± 6.68	156.66 ± 5.53	<**0.001^b^**	160.13 ± 6.36	156.20 ± 6.55	0.061^b^		
BMI	25.40 ± 3.48	23.06 ± 2.61	**0.001^b^**	23.18 ± 3.37	24.08 ± 2.95	0.285^b^		
BSA, m^2^	1.72 ± 0.15	1.57 ± 0.10	<**0.001^b^**	1.62 ± 0.11	1.59 ± 0.11	0.325^b^		
Preoperative TSH, mIU/L	3.27 ± 1.74	1.76 ± 1.41	**<0.001^b^**	3.57 ± 2.10	1.67 ± 1.31	**0.010** ^b^	**0.013**	0.490(0.278,0.861)
Preoperative FT3, pmol/L	8.37 ± 5.47	4.74 ± 1.22	**<0.001^b^**	8.13 ± 5.63	5.18 ± 1.72	0.089^b^		
Preoperative FT4, pmol/L	11.28 ± 6.88	16.06 ± 3.40	**0.004^b^**	11.71 ± 7.38	15.78 ± 4.14	0.367^b^		

Variables with statistical significance are shown in bold. Weight and mean temperature were matched between groups. ^a^ chi-square test, ^b^ Mann−Whitney test. p* was the result of binary regression analysis.

## 4 Discussion

For patients with an intermediate to high risk of recurrence, suppression of TSH is required to reduce the risk of recurrence (22–24). In elderly patients, it is essential to balance the benefits of suppression treatment with the side effects of iatrogenic hyperthyroidism. At present, the factors influencing LT4 medication based on age stratification remain unclear. In this study, we analyzed the factors influencing postoperative LT4 medication based on age in PTC patients with intermediate to high risks of recurrence, which has important value for the formulation of personalized LT4 medication regimens.

Consistent with a previous study, we observed that several factors affected the treatment effect, such as ambient temperature and BMI. Since thyroid hormone is required for normal development as well as regulating metabolism in the adult (25, 26), previous studies usually focused on the effects of body weight (27, 28). In our study, the patients all took LT4 at an empirical regimen of 1.6 µg/kg, and more than 70% did not achieve the expected TSH suppression level. This suggests that there are many factors other than body weight that affect the effect of LT4. Ambient temperature is an important factor affecting the body’s metabolism. Therefore, our study considered the influence of ambient temperature on the effect of LT4 medication. We used BW and ambient temperature in the calculation of the propensity score to minimize their impact on the results. Surprisingly, although the effects of weight and ambient temperature were diminished, age was still an independent factor that affected the treatment effect of suppressing TSH levels among patients with an intermediate to high risk of recurrence.

It has been proven that the homeostasis of the endocrine system changes with the advancement of age, as does thyroid hormone regulation. The suppression of TSH by thyroid hormone is greater in elderly individuals (7, 29), which means that under a state of hypothyroidism, elderly individuals have a smaller rise in TSH concentration. In other words, a given dose suppresses the secretion of TSH more with advancing age. Age-related differences in treatment outcomes may be associated with changes in the hypothalamic-pituitary-thyroid axis in different organ hierarchies (20). For such changes, there is no specific age at which the effect begins. Several previous studies indicated that 97.5th percentile of serum TSH was noted to increase by 0.1-0.3 mU/L every 10 years after 30~39 years of age (21, 30, 31). The functional decline of the thyroid gland, causing it to secrete less thyroid hormone, could be the cause of upper reference limit of TSH in elderly individuals. The longer period of elimination of thyroid hormone in elderly individuals seems to result in a better suppression effect of LT4 (32).

Preoperative TSH was an independent factor in both elderly and younger patients after we stratified patients based on age. Our results showed that patients with lower preoperative TSH levels are more likely to achieve a state of TSH suppression after surgery. We suspect that variable changes in the sensitivity or numbers of thyroid hormone receptors in the pituitary and hypothalamus explain this correlation. 

The preoperative fT3 level was also shown to be an independent factor among patients under 55 years old. In our study, patients with higher preoperative fT3 were less likely to suppress the secretion of TSH, which may be the result of the adaptation of the pituitary to higher fT3. An interesting correlation between presurgical fT3 and the treatment effect was also seen in Vincenzo Di Donna’s study (11). This result indicates that iodothyronine deiodinases may also function in the suppression of TSH secretion. Since the secretion of TSH is suppressed by fT4 and fT3, the meaning of fT3 or TSH as a predictor may be the same. It is also interesting that fT3 did not show significance among patients aged 55 and over, which is expected due to their decreased metabolism of thyroid hormone (33, 34) or the changes in the sensitivity of the hypothalamic-pituitary-thyroid axis during aging.

Previous studies have shown that age-related thyroid changes differ by sex (35) and sex was indicated to be relative with lymph node metastasis in PTC (36). So, is the age-related level of TSH suppression after LT4 medication also related to sex? In our analysis, sex was not an independent influencing factor for either people aged 55 years older or those younger than 55 years old. We considered that this was because sex hormones have little effect on the response to thyroid hormones in the hypothalamus and pituitary gland.

Even though sex was not identified as an independent factor affecting the TSH suppression level, it was shown to be a factor that led to a worse treatment effect. Among patients with unexpected levels, men were more likely to have TSH levels beyond the physiological range. This means that men require a larger LT4 dose if they cannot suppress TSH in the first month after surgery on the usual dose.

There are some limitations in our study. First, although we tried to determine the relationship between treatment outcome and some comorbidities, the number of patients who had comorbidities was too small and even smaller after PSM to reveal any relationship. In addition, patient adherence to LT4 is uncertain, which may bias our results. In our study, we only tried to determine the predictors of TSH suppression degree. It remains to be studied further how to adjust the initial levothyroxine dose according to known factors to obtain a satisfactory targeting suppression degree.

## 5 Conclusion

For PTC patients with intermediate to high risk of recurrence, age is an independent risk factor for achieving the target TSH levels through usual dosage of LT4. Under the condition of the same body weight and ambient temperature, it is easier to achieve the target TSH suppression level in patients aged less than 55 years old with lower preoperative TSH levels and fT3 and in patients aged 55 and older with lower preoperative TSH levels (**Table 6**).

**Table 6:** Identified factors leading to higher LT4 requirement to achieve suppression TSH level.

factors	OR(95%CI)	
	<55 years old	≥55 years old
preoperative fT3	0.859(0.746,0.990)	
preoperative TSH	0.588(0.459,0.753)	0.490(0.278,0.861)

## Data availability statement

The raw data supporting the conclusions of this article will be made available by the authors, without undue reservation.

## Ethics statement

This study received approval from the medical ethical committee at Xiangya Hospital (20211245).Written informed consent to participate in this study was waived because of the retrospective and anonymous nature of the study.

## Author contributions

Study concepts, R-YZ and PH. Study design, PH and SC. Data acquisition, LA, NT, and NL. Quality control of data and algorithms, PC, ML, and D-JO-Y. Data analysis and interpretation, LA, BW, and Y-XZ. Statistical analysis, Z-EQ and H-LT. Manuscript preparation, R-YZ and PH. Manuscript editing, R-YZ and PH. Manuscript review, PH and SC. All authors contributed to the article and approved the submitted version.
